# Could herbal soup be a potentially unrecognized cause of hepatotoxicity at autopsy?

**DOI:** 10.1007/s12024-022-00490-5

**Published:** 2022-06-24

**Authors:** Susan M. Britza, Rachael Farrington, Ian F. Musgrave, Craig Aboltins, Roger W. Byard

**Affiliations:** 1 Adelaide Medical School, The University of Adelaide, Level 2 Helen Mayo North Building, Frome Road, Adelaide, SA 5005 Australia; 2 Department of Infectious Diseases, The University of Melbourne, Melbourne, VIC 3010 Australia; 3 Forensic Science South Australia, Adelaide, SA 5000 Australia

**Keywords:** Herbal soup, *Bak kut the*, Hepatotoxicity, Idiosyncratic response, In vitro culture

## Abstract

Unexpected hepatic failure with liver necrosis is sometimes encountered during a forensic autopsy. Determining the etiology may sometimes be difficult, although increasingly herbal medicines are being implicated. To determine whether such effects might also be caused by foodstuffs, the following in vitro study was undertaken. Four formulations of traditional herbal soup advertised as *bak kut teh* were prepared and added to cultures of liver carcinoma cells (HepG2). Cell viability was assessed using an MTT colorimetric assay at 48 h demonstrating that all formulations had significant toxicity prior to dilution (*p* < 0.05). Formulation #1 showed 21% cell death (*p *= 0.023), Formulation #2 30% (*p *= 0.009), and Formulation #3 41% (*p *< 0.0001). Formulations #1–3 showed no significant toxicity once diluted (*p *> 0.05). Formulation 4 showed approximately 83% cell death before dilution (*p *< 0.0001) and persistent toxicity even with dilutions at 1:10 (15% ± 3.7, *p *= 0.023) and 1:1000 (14% ± 3.8, *p* = 0.024). This study has shown that herbal foodstuffs such as *bak kut teh* may be responsible for variable degrees of in vitro hepatotoxicity, thus extending the range of herbal products that may be potentially injurious to the liver. If unexpected liver damage is encountered at autopsy, information on possible recent ingestion of herbal food preparations should be sought, as routine toxicology screening will not identify the active components. Liver damage may therefore be caused not only by herbal medicines but possibly by herbal products contained in food.

## Introduction

Herbal medicines have been used for many thousands of years in communities and cultures around the world, and particularly in Asia, to treat a myriad of conditions. In recent years, there has been a marked increase in their use in Western countries with China exporting more than $500 million worth of traditional Chinese medicines to the USA in 2016 alone [1, 2]. The reasons for this are complex; however, an underlying belief is that as herbal products are natural, they must therefore be much safer to use than manufactured pharmaceutical agents [3]. This position fails to acknowledge that any agent that has therapeutic effects must also have the potential for therapeutic side effects.

Ongoing reports in the literature have detailed cases where lethal events have involved hepatic and renal failure due to the effects of individual herbal agents or to the interaction of herbal agents with other herbs or with prescribed drugs. Polyherbacy is an important area to study as the pharmacokinetics of herbal preparations are often poorly understood [4, 5].

The potential for dietary herbs to cause toxicity has not, however, been recognized or previously investigated. The following in vitro study was undertaken to evaluate whether *bak kut teh* could also have potential hepatotoxic effects that might be responsible for significant liver damage identified at autopsy.

## Materials and methods

Four formulations of herbal soup advertised as *bak kut teh* were randomly selected and purchased from retailers or health food stores. Each formulation was de-identified and prepared following the package instructions to create a soup. This consisted of adding a sachet of soup mix to boiling water for 5 min. Formulations were then further diluted using PBS to a final dilution factor of 1:10,000–1. Standard laboratory methodology was used with exposure to cultures of liver carcinoma cell line (HepG2) to the *bak kut teh* formulations. The HepG2 cell line has been extensively used in our laboratory as an appropriate in vitro model for liver tissue. Cell viability was assessed using an MTT colorimetric assay according to standard procedures [6], with statistical significance determined via one-way analysis of variance (ANOVA).

## Results

Details of the ingredients of each of the soups varied considerably in detail from one preparation label to another. The ingredients included the following: Formulation #1, dried hawthorn; Formulation #2, goji berries, ginseng, bark, and dried mushrooms; Formulation #3, astragalus, Polygonatum odoratum, Ligusticum chuanxiong, Codonopsis pilosula, Cinnamomum cassia, Angelica sinensis, Illicium verum, Piper nigrum, and Eugenia caryophyllata; and Formulation #4, spices, pepper, and salt.

**Fig. 1:**
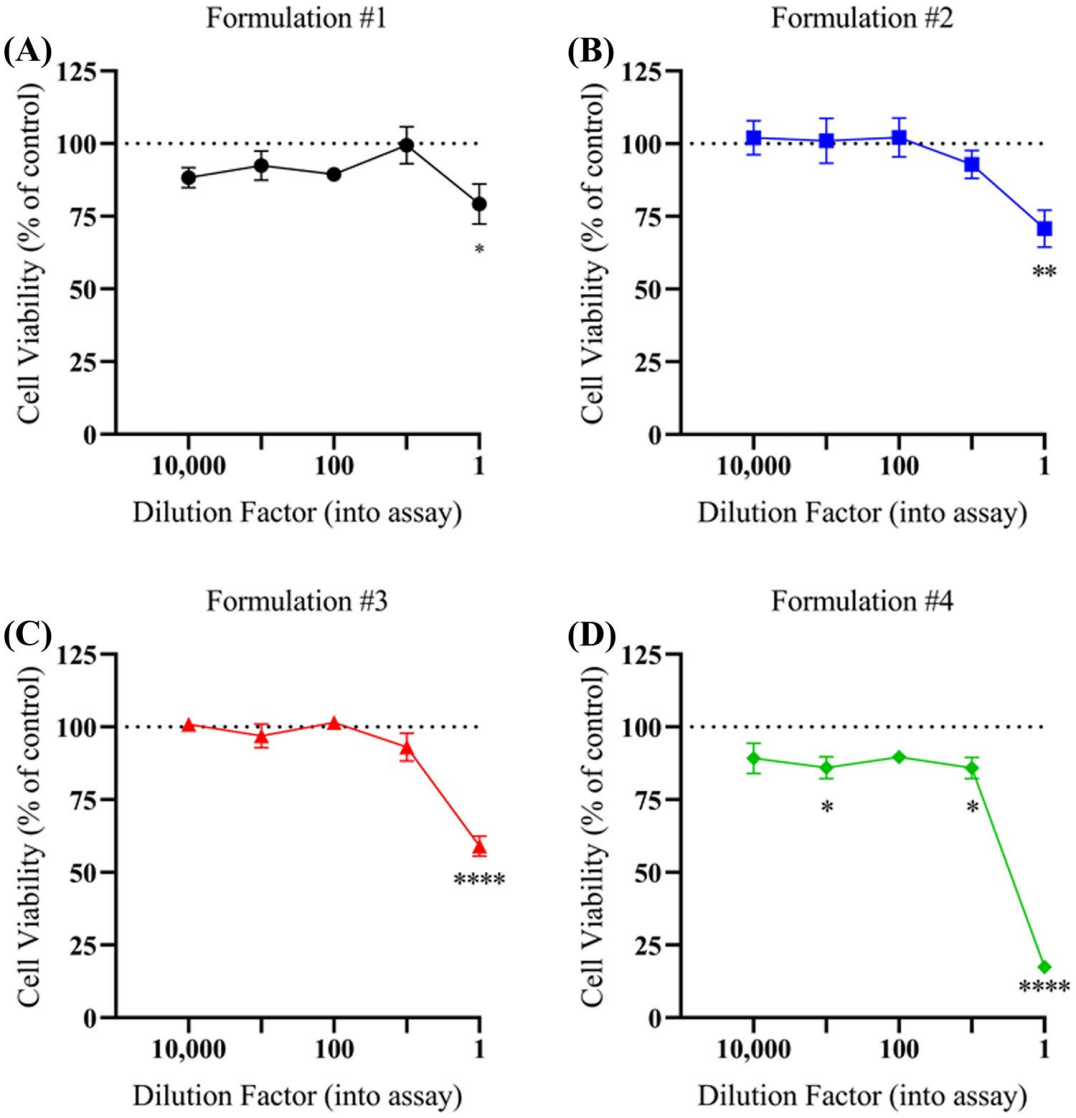
The effect of four different *bak kut teh* formulations on HepG2 cells after 48 h of exposure. **A** Formulation #1 showed significant toxicity before dilution (*p *= 0.023), but demonstrated no significant toxicity once diluted (*p *> 0.05). **B** Formulation #2 demonstrated significant toxicity with no dilution factor (*p *= 0.009), but none once diluted (*p *> 0.05). **C** *Bak kut teh* Formulation #3 showed similar toxicity when undiluted (*p *< 0.0001) but was not found to be toxic with dilution (*p* > 0.05). **D** Formulation #4 was shown to be significantly toxic undiluted (*p *< 0.0001) and in dilutions with a factor of 1:10 (*p* = 0.023) and 1:1000 (*p *= 0.024). Values mean ± SEM (*n* = 3). *p < 0.05, **p < 0.01, ****p < 0.0001. One-way ANOVA with Dunnett’s. Where error bars are not shown, they are smaller than the symbol

However, all formulations of *bak kut teh* demonstrated significant toxicity (*p* < 0.05). Formulation 1 showed the least toxic response, with approximately 21% cell death observed (*p *= 0.023), followed by Formulation 2 with 30% (*p* = 0.009) and Formulation 3 with 41% (*p* < 0.0001). Formulations 1–3 showed no significant toxicity once diluted (*p* > 0.05) (Fig. 1A–C). Formulation 4 showed the most significant toxicity to the HepG2 cell line with approximately 83% cell death before dilution (*p* < 0.0001) and persistent toxicity even with dilution 1:10 (15% ± 3.7, *p *= 0.023) and 1:1000 (14% ± 3.8, *p* = 0.024) (Fig. 1D). Control cell cultures without *bak kut teh* showed no cell death.

## Discussion

The dangers of herbal preparations relate to issues of inherent toxicity, replacement of active agents by other plants, augmentation with undisclosed pharmaceutical drugs, and interactions with other herbs and drugs [7]. In addition, idiosyncratic metabolic responses may render certain individuals more susceptible to negative herb and herb-drug effects. The variability in content of the active ingredients from batch to batch of a particular preparation and between different preparations is another issue to consider, a factor which may also arise with herbal foodstuffs.

A recent study by our group demonstrated an exacerbation of the potential of paracetamol to cause damage to liver cells (in this case also grown in culture) in the presence of psoralen, a furanocoumarin compound that is present in *Psoralea corylifolia*, a common Chinese herb [6]. The potential for herbal agents to increase paracetamol hepatotoxicity is of particular concern in the era of COVID-19 when paracetamol may be the drug of choice to treat virus-associated fevers and myalgias with concomitant herbal preparations being promoted as beneficial [8–10]. We have also demonstrated injury to intestinal epithelial cells from herbal preparations [11].

Although adverse clinical reactions to *bak kut teh* are not reported, one of the authors (CA) has had a patient who presented with toxic symptoms including rhabdomyolysis 2 days after consuming the soup. As we have an established system of hepatocyte cell culture that has been shown to be useful in assessing herbal toxicity, we therefore decided to expose HepG2 cells to *bak kut teh* formulations. The results clearly demonstrated quite significant hepatocyte toxicity in four preparations at concentrations normally found in the soup broth. Of particular concern was Formulation #4 where significant hepatotoxicity continued to occur even at dilutions of 1/1000. As with herbal therapies, another issue involved the variable declaration of ingredients, with one merely listing “spices, pepper, salt” compared to another which had an extensive list including Astragalus, Polygonatum odoratum, Ligusticum chuanxiong, Angelica sinensis, and Eugenia caryophyllata. Variations in effect could however come from different volumes of liquid being used during the boiling of the herbal sachets and also from natural dilution of the soup within the stomach, both of which may alter the risk of toxicity.

In conclusion, this study has shown that not only herbal therapeutic agents may be responsible for in vitro hepatotoxicity but that this may also extend to herbal foodstuffs such as *bak kut teh*. This result should not perhaps come as a surprise, as a number of herbal products may interfere with a variety of metabolic pathways, and this should not necessarily exclude herbal material that is being used in food. This study has extended, therefore, the range of herbal products that may be potentially dangerous to health and should prompt assessment of dietary intake of herbal soups in cases that present to autopsy with sometimes fulminant liver failure and/or hepatic necrosis. Further study will clearly be required to evaluate this potential association; however, it may be of particular significance to individuals with pre-existing liver disease.

## Key points

1. To determine whether hepatotoxicity might be caused by herbal foodstuffs the following in vitro study was undertaken.
2. Four formulations of traditional herbal soup advertised as “bak kut teh” were added to cultures of liver carcinoma cells (HepG2).
3. All formulations were responsible for variable degrees of in vitro hepatotoxicity.
4. Liver damage may therefore be caused not only by herbal medicines but possibly by herbal products contained in food.
